# Neuroethics of Non-primary Brain Computer Interface: Focus on Potential Military Applications

**DOI:** 10.3389/fnins.2018.00696

**Published:** 2018-10-23

**Authors:** Charles N. Munyon

**Affiliations:** Department of Neurosurgery, Lewis Katz School of Medicine, Temple University Hospital, Philadelphia, PA, United States

**Keywords:** brain-computer interface, military, restorative, neurostimulation, augmentative

## Abstract

The field of neuroethics has had to adapt rapidly in the face of accelerating technological advancement; a particularly striking example is the realm of Brain-Computer Interface (BCI). A significant source of funding for the development of new BCI technologies has been the United States Department of Defense, and while the predominant focus has been restoration of lost function for those wounded in battle, there is also significant interest in augmentation of function to increase survivability, coordination, and lethality of US combat forces. While restoration of primary motor and sensory function (primary BCI) has been the main focus of research, there has been marked progress in interface with areas of the brain subserving memory and association. Non-Primary BCI has a different subset of potential applications, each of which also carries its own ethical considerations. Given the amount of BCI research funding coming from the Department of Defense, it is particularly important that potential military applications be examined from a neuroethical standpoint.

## Introduction

Military imperatives have driven medical advances and allowed for rapid implementation and evaluation of new practices (Beekley et al., 2007; Haider et al., 2015). Many of these advances can then be applied to civilian practices, yielding additional dividends on the often considerable investment underpinning their development. The development of resuscitative endovascular balloon occlusion of the aorta, for example, shows how dedication of the military’s resources to rapidly solving an urgent problem (in this case battlefield fatalities due to non-compressible thoracic or pelvic bleeding) can significantly accelerate the development and deployment of new technologies for wartime and peacetime applications (Rasmussen and Eliason, 2017). The field of neuroprosthetics and brain computer interface (BCI) is another area where a significant funding pool has come from Department of Defense sources, in particular the Defense Advanced Research Projects Agency (DARPA) (Miranda et al., 2015). This presents an area of significant opportunity for national defense and also human neuroscience; it also raises several potential ethical issues that should be explored before the technology has already transitioned to the domain of military application.

In their review of military applications for BCI in 2010, Kotchetkov et al. (2010) focused on restoration or augmentation of function using primary motor or sensory pathways. They also proceeded from the reasonable assumption that the flow of information would be in one direction: from brain to machine decoder and from there to the executor of the desired effect. The adoption of responsive neurostimulation for epilepsy, however, demonstrated that a self-contained neuromodulation system can both analyze and modify brain activity via a skull-mounted processor (Gigante and Goodman, 2011). There has been significant progress in transcranial stimulation paradigms for non-invasive cortical and sub-cortical neuromodulation (Nelson et al., 2016; Giordano et al., 2017). The addition of MRI-based navigation to transcranial magnetic stimulation has allowed for precise non-invasive cortical mapping and targeted treatment (Wu et al., 2014). Advances in functional network mapping (Craddock et al., 2012) and elucidation of the relative roles of the salience, default-mode, and central-executive networks in input processing (Chand and Dhamala, 2016), as well as cortico-limbic pathways in affect regulation (Mincic, 2015) and decision making (Onge et al., 2012) have significantly accelerated the timeline for bidirectional BCI targeting substrates outside of the primary motor and sensory networks (non-primary brain computer interface, or NpBCI).

## Neuroethical Framework

As the rate of development accelerates, it is important that neuroethics not lag behind. In particular, technologies developed under projects with Department of Defense Funding or having an obvious military application should receive enhanced scrutiny due to the differences between civilian and military medical ethics. Unfortunately, there have been recent instances where physicians affiliated with defense or intelligence agencies have (erroneously) interpreted the principle of “dual loyalty” as allowing for significant deviations from Hippocratic principles in the name of National Security (Rubenstein and Xenakis, 2010; Balfe, 2016), and the best way to prevent similar problems with application of NpBCI is to ensure that through robust discussion, boundaries can be demarcated before they have an opportunity to be crossed. To facilitate that discussion, I am proposing a framework for classification of these technologies within one of three categories: restorative, augmentative, or disruptive. The therapies within each category can then be examined in terms of their risk to the subject, with different acceptable risk/benefit ratios depending on the category.

The primary assumption in civilian medical BCI research is that technology is being developed to either restore lost function or prevent further deterioration of function in the face of a disease process or injury; this “restorative” paradigm will likely predominate in both civilian and military medical research. Significant discussion has already taken place, however, regarding BCI techniques for augmentation of function in neurologically normal individuals. The term “cosmetic neurosurgery” was introduced by Lipsman and Lozano (2015) to describe the use of neuromodulation for non-therapeutic purposes. In the case of military applications, “augmentative” is a potentially more appropriate descriptor than “cosmetic,” as the primary purpose of this technology would be to augment the lethality, survivability, or efficiency of military personnel. Finally, consideration must be given to ways in which BCI could be used to disrupt individual autonomy for purposes such as interrogation or pacification. This “disruptive” category must be evaluated not only to ensure that a robust ethical framework exists for any use by American and allied agencies, but also so that countermeasures can be considered in the event of adoption by America’s adversaries.

## Considerations for Individual Categories

### Restorative

Most current BCI technologies fall under the category of restorative, and this will likely remain true for the foreseeable future, particularly as regards invasive therapies. Development of restorative NpBCI technologies for military use will likely focus on amelioration of post-traumatic memory or mood disturbances (eg., Miller et al., 2015; Gagnepain et al., 2017), and will generally involve essentially the same ethical analysis as would be employed on the civilian side. For example, questions of autonomy and acceptable risk would be unlikely to differ between wounded service members and civilians. Questions of distributive justice, however, may differ slightly, insofar as America is generally tolerant of a higher spending threshold for those wounded in military service. Prior to development, consideration should be given to whether any technology could be easily adapted for disruptive applications, but provided that the problem being addressed is clinically important, continuation of the project is likely to be justifiable unless the potential for misuse is particularly egregious.

### Augmentative

Augmentative applications for BCI could include enhancement of native faculties, suppression of states that interfere with optimal performance, or facilitation of communication between a combatant and their human team-mates or non-human support infrastructure. Enhancing lethality, improving survivability, and maintaining combat readiness are primary imperatives in training and maintenance of a warfighting force, and considerations regarding privacy and bodily autonomy can function differently in a military context. Any intervention that would incur more than minimal risk will require intensive ethical examination, and risk to the subject should be considered beyond the framework of invasiveness or surgical risk given the potential for modification of limbic or executive function. Freedom of consent may be challenging given the presence of a hierarchical command structure, as well as internal pressure that soldiers may feel to adopt any technology which might improve survivability for themselves and their teammates. Use of npBCI for threat identification, reaction time augmentation, or distributed decision-making applications may change emotional and psychological response to the intended (target neutralization) and unintended (civilian or “friendly fire” casualty) consequences of lethal force; downstream mental health effects of these changes are difficult to predict, but must be monitored prospectively and proactively. Consideration should also be given to the ease with which any augmentative NpBCI system designed for battlefield use could be breached, reverse-engineered, or subverted for disruptive use by an adversary.

### Disruptive

Unlike restorative or augmentative applications, where primary BCI is significantly in advance of NpBCI in terms of available technology, realistic uses for disruptive NpBCI are significantly closer to feasibility than for primary BCI. There has been significant progress in the development of reliable electroencephalographic or near-infrared markers for deception or dissimulation (Abootalebi et al., 2006; Abootalebi et al., 2009; Sai et al., 2014a,b, 2016). Non-invasive BCI technology for detection of deception shows greater portability and ease of use compared to functional neuroimaging for the same ends. Ethical analysis of forensic neuroimaging in the literature (Hyman, 2010; Moreno and Parashar, 2012) can be broadly applied to non-invasive BCI; within the psychophysiological literature regarding EEG-based forensic techniques, however, the main consideration has been admissibility in US court rather than ethics (Rosenfeld et al., 2013; Kraft and Giordano, 2017). There is no current international consensus on whether an individual enjoys any expectation of electrocerebral privacy, and only recently has there been any call to apply protections to electrocerebral or neuroimaging data similar to those in place for genomic data (Ienca and Andorno, 2017). This lack of consensus means that non-invasive, unidirectional NpBCI as an adjunct to interrogation will likely remain a gray area unless significant attention is paid to the question. Disruptive stimulation paradigms are ethically much harder to countenance. Non-invasive transcranial stimulation without the subject’s consent may have a therapeutic role in cases of intractable aggression or similar disorders, but non-consensual non-invasive stimulation for purposes of pacification, interrogation, or torture (i.e., stimulation of somatosensory areas to induce pain without physical trauma, or suppressive stimulation of dorsal neocortical structures to induce psychological distress) is clearly impermissible. Particularly concerning is the potential for closed-loop coupling of the detection of deception or dissimulation with noxious transcranial stimulus, creating a powerful tool for interrogation without infliction of physical trauma. As military ethical guidelines (Annas, 2008) make clear, any surgeon implanting a device for disruptive neuromodulation in a military setting would be in violation of both the tenets of medical ethics and the Geneva Conventions. The potential for adoption of disruptive technology by adversarial state or non-state actors should also promote significant reflection prior to undertaking any such project, as well as consideration regarding possible countermeasures.

From a geostrategic standpoint, it is worth noting that much of the work on BCI-based detection of deception or dissimulation has been performed at state sponsored academic institutions in Iran and China. This should be a cause for potential concern, particularly given the differences in understanding of personal freedom and autonomy between our cultures. The performance of ablative surgeries for mental illness in China without clear consent further highlights the potential danger that BCI technology could be abused by an authoritarian state (Zamiska, 2007; Wu et al., 2010; Zhang et al., 2017).

## Discussion

As Brain Computer Interface becomes less invasive and more sophisticated, applications have already started to expand outside of the medical sphere and into the realm of consumer technology. Potential military applications also continue to advance, and in an environment where research funding in increasingly harder to obtain, the Department of Defense can be an important source of support. The fundamental mission of the Department of Defense differs significantly from that of the National Institutes of Health, however, and these differences should prompt additional scrutiny from both basic and translational researchers. In this paper, I propose an ethical framework to facilitate discussion, beginning with the classification of a technology into one of three domains: restorative, augmentative, or disruptive. Consideration should be given as to whether the technology has applications across domains: for example, restorative NpBCI for patients with PTSD could also potentially augment warfighters’ capabilities to modulate their response to stressful stimuli on the battlefield; this phase of analysis should consider how difficult it would be to repurpose the technology, and thus how likely it would be that it would be used outside of its intended scope. Finally, the analysis should include the likely risks to patient physical health, emotional well-being, and bodily autonomy. These considerations will be predominantly related to the invasiveness of the technology, but as discussed previously, will also be concerned with alterations in normal emotional, or cognitive function. Within the domain of disruptive BCI, where some disruption of autonomy is a given, a significant conversation will need to be held regarding what kinds of interventions would or would not be acceptable, under what circumstances, and what mechanisms might be available for monitoring appropriate use.

The framework proposed here is obviously not exhaustive, but is intended to provoke discussion in a way that could help ensure that the pace of technological advancement does not too far outstrip our ethical consensus, particular in the domain of National Security, where ethical considerations can differ substantially from those of civilian medical research. Such discussion could help ensure that medical researchers and physicians maintain a seat at the table regarding application of the technology that they help develop, and could also spark new ideas for innovations.

## Author Contributions

CM was responsible for the conceptualization, research, and preparation of this article.

## Disclaimer

Any opinions expressed in this piece belong solely to the author and should not be attributed in any way to the Department of Defense, the Department of the Army, or any other branch of the Federal Government. All information was drawn from publicly available sources, and the author does not have any access to or knowledge of classified material related to the concepts discussed in this commentary.

## Conflict of Interest Statement

The author does hold the rank of Major in the United States Army Reserve Medical Corps, but this analysis was written in a civilian capacity and without input or compensation from the Department of Defense or any other Federal entity.

## References

[B1] Abootalebi V. Moradi M. H. Khalilzadeh M. A. (2006). A comparison of methods for ERP assessment in a P300-based GKT. *Int. J. Psychophysiol.* 62 309–320. 10.1016/j.ijpsycho.2006.05.009 16860894

[B2] Abootalebi V. Moradi M. H. Khalilzadeh M. A. (2009). A new approach for EEG feature extraction in P300-based lie detection. *Comput. Methods Prog. Biomed.* 94 48–57. 10.1016/j.cmpb.2008.10.001 19041154

[B3] Annas G. (2008). Military medical ethics-physician first, last, always. *N. Engl. J. Med.* 359 1087–1087. 10.1056/NEJMp0805975 18784096

[B4] Balfe M. (2016). Standardizing psycho-medical torture during the War on Terror: why it happened, how it happened, and why it didn’t work. *Soc. Sci. Med.* 171 1–8. 10.1016/j.socscimed.2016.11.014 27855322

[B5] Beekley A. C. Starnes B. W. Sebesta J. A. (2007). Lessons learned from modern military surgery. *Surg. Clin. North Am.* 87 157–184. 10.1016/j.suc.2006.09.008 17127127

[B6] Chand G. Dhamala M. (2016). Interactions among the brain default-mode, salience, and central-executive networks during perceptual decision-making of moving dots. *Brain Connect.* 6 249–254. 10.1089/brain.2015.0379 26694702

[B7] Craddock R. C. James G. A. Holtzheimer P. E. Hu X. P. Mayberg H. S. (2012). A whole brain fMRI atlas generated via spatially constrained spectral clustering. *Hum. Brain Mapp.* 33 1914–1928. 10.1002/hbm.21333 21769991 PMC3838923

[B8] Gagnepain P. Hulbert J. Anderson M. (2017). Parallel regulation of memory and emotion supports the suppression of intrusive memories. *J. Neurosci.* 37 6423–6423. 10.1523/JNEUROSCI.2732-16.2017 28559378 PMC5511877

[B9] Gigante P. R. Goodman R. R. (2011). Responsive neurostimulation for the treatment of epilepsy. *Neurosurg. Clin. N. Am.* 22 477–480. 10.1016/j.nec.2011.07.003 21939846

[B10] Giordano J. Bikson M. Kappenman E. Clark V. Coslett H. Hamblin M. R. (2017). Mechanisms and effects of transcranial direct current stimulation. *Dose Response* 15:1559325816685467. 10.1177/1559325816685467 28210202 PMC5302097

[B11] Haider A. Piper L. Zogg C. Schneider E. Orman J. (2015). Military-to-civilian translation of battlefield innovations in operative trauma care. *Surgery* 158 1686–1686. 10.1016/j.surg.2015.06.026 26210224

[B12] Hyman S. (2010). Emerging neurotechnologies for lie-detection: where are we now? An appraisal of Wolpe, Foster and Langleben’s “Emerging neurotechnologies for lie-detection: promise and perils” five years later. *Am. J. Bioeth.* 10 49–50. 10.1080/15265161.2010.527263 20945267

[B13] Ienca M. Andorno R. (2017). Towards new human rights in the age of neuroscience and neurotechnology. *Life Sci. Soc. Policy* 13:5. 10.1186/s40504-017-0050-1 28444626 PMC5447561

[B14] Kotchetkov I. S. Hwang B. Y. Appelboom G. Kellner C. P. Connolly E. S. Jr. (2010). Brain-computer interfaces: military, neurosurgical, and ethical perspective. *Neurosurg. Focus* 28:E25. 10.3171/2010.2.FOCUS1027 20568942

[B15] Kraft C. Giordano J. (2017). Integrating brain science and law: neuroscientific evidence and legal perspectives on protecting individual liberties. *Front. Neurosci.* 11:621. 10.3389/fnins.2017.00621 29167633 PMC5682320

[B16] Lipsman N. Lozano A. (2015). Cosmetic neurosurgery, ethics, and enhancement. *Lancet Psychiatry* 2 585–586. 10.1016/S2215-0366(15)00206-0 26303544

[B17] Miller J. P. Sweet J. A. Bailey C. M. Munyon C. N. Luders H. O. Fastenau P. S. (2015). Visual-spatial memory may be enhanced with theta burst deep brain stimulation of the fornix: a preliminary investigation with four cases. *Brain* 138(Pt 7), 1833–1842. 10.1093/brain/awv095 26106097

[B18] Mincic A. M. (2015). Neuroanatomical correlates of negative emotionality-related traits: a systematic review and meta-analysis. *Neuropsychologia* 77 97–118. 10.1016/j.neuropsychologia.2015.08.007 26265397

[B19] Miranda R. Casebeer W. Hein A. Judy J. Krotkov E. Laabs T. L. (2015). DARPA-funded efforts in the development of novel brain-computer interface technologies. *J. Neurosci. Methods* 244 52–67. 10.1016/j.jneumeth.2014.07.019 25107852

[B20] Moreno J. Parashar S. (2012). “National security, brain imaging, and privacy,” in *I Know What You’re Thinking*, eds Richmond S. Rees G. Edwards S. (Oxford: Oxford University Press), 173–180. 10.1093/acprof:oso/9780199596492.003.0013

[B21] Nelson J. McKinley R. A. Phillips C. McIntire L. Goodyear C. Kreiner A. (2016). The effects of transcranial direct current stimulation (tDCS) on multitasking throughput capacity. *Front. Hum. Neurosci.* 10:589. 10.3389/fnhum.2016.00589 27965553 PMC5126079

[B22] Onge S. Stopper C. Zahm D. Floresco S. (2012). Separate prefrontal-subcortical circuits mediate different components of risk-based decision making. *J. Neurosci.* 32 2886–2899. 10.1523/JNEUROSCI.5625-11.2012 22357871 PMC3609657

[B23] Rasmussen T. E. Eliason J. L. (2017). Military-civilian partnership in device innovation: development, commercialization and application of resuscitative endovascular balloon occlusion of the aorta. *J. Trauma Acute Care Surg.* 83 732–735. 10.1097/TA.0000000000001661 28930964

[B24] Rosenfeld J. P. Hu X. Labkovsky E. Meixner J. Winograd M. R. (2013). Review of recent studies and issues regarding the P300-based complex trial protocol for detection of concealed information. *Int. J. Psychophysiol.* 90 118–134. 10.1016/j.ijpsycho.2013.08.012 24012907

[B25] Rubenstein L. Xenakis S. (2010). Roles of CIA physicians in enhanced interrogation and torture of detainees. *JAMA* 304 569–570. 10.1001/jama.2010.1057 20682939

[B26] Sai L. Lin X. Hu X. Fu G. (2014a). Detecting concealed information using feedback related event-related brain potentials. *Brain Cogn.* 90 142–150. 10.1016/j.bandc.2014.06.012 25058495

[B27] Sai L. Lin X. Rosenfeld J. P. Sang B. Hu X. Fu G. (2016). Novel, ERP-based, concealed information detection: combining recognition-based and feedback-evoked ERPs. *Biol. Psychol.* 114 13–22. 10.1016/j.biopsycho.2015.11.011 26638760

[B28] Sai L. Zhou X. Ding X. Fu G. Sang B. (2014b). Detecting concealed information using functional near-infrared spectroscopy. *Brain Topogr.* 27 652–652. 10.1007/s10548-014-0352-z 24514911

[B29] Wu H. M. Wang X. L. Chang C. W. Li N. Gao L. Geng N. (2010). Preliminary findings in ablating the nucleus accumbens using stereotactic surgery for alleviating psychological dependence on alcohol. *Neurosci. Lett.* 473 77–81. 10.1016/j.neulet.2010.02.019 20156524

[B30] Wu S. W. Maloney T. Gilbert D. L. Dixon S. G. Horn P. S. Huddleston D. A. (2014). Functional MRI-navigated repetitive transcranial magnetic stimulation over supplementary motor area in chronic tic disorders. *Brain Stimul.* 72 212–218. 10.1016/j.brs.2013.10.005 24268723

[B31] Zamiska N. (2007). In China, brain surgery is pushed on the mentally ill. *Wall St. J.* 1–10.

[B32] Zhang S. Zhou P. Jiang S. Li P. Wang W. (2017). Bilateral anterior capsulotomy and amygdalotomy for mental retardation with psychiatric symptoms and aggression: a case report. *Medicine* 96:e5840. 10.1097/MD.0000000000005840 28072743 PMC5228703

